# Macon Medical Society

**Published:** 1894-08

**Authors:** 


### *SOCIETY REPORTS.*

### MACON MEDICAL SOCIETY.

MACON, GA., April 3, 1894.

*Dr. A. C. Blain, President, in the chair.*

DR. N. G. GEWINNER, essayist for the evening, read a paper on the "Therapeutics of Infancy and Childhood." See page 337.

### DISCUSSION.

DR. K. P. MOORE said that he had been interested in the excellent paper of Dr. Gewinner, and he could do no more than emphasize some of the thoughts suggested in the paper. Speaking of the so-called summer complaints of children, and the teething period, there is no doubt but that a large part of them have their origin and etiology in the *one* source, viz., inprudence in feeding. That the teething may play some minor part in the gastro-intestinal disturbances of infancy there is scarcely any doubt, but it is a settled question by a large array of modern authors on diseases of children that the secret of almost all these disturbances lies in a want of proper care and regard for the feeding of infants. In the foundling institutions, and in private practice where intelligent care and forethought can be properly carried out, the teething period is no longer looked upon as the great nightmare of the infant's life, and we no longer feel that it is necessary that a child should go through several long months of the trying ordeal of cutting its teeth. There is one point in the treatment of these gastro-intestinal disturbances which was not fully brought out in the paper of Dr. Gewinner which I would like to emphasize, viz., the use of water at both ends of the alimentary tract. I have seen some wonderfully good results in washing out the stomach and rectum and colon with simple warm water. Can now recall several instances in one of the New York foundling hospitals, and in private practice, where the stomach was so irritable as not to be able to retain even a teaspoonful of the *very simplest* diet, relieved almost like magic and peaceful and refreshing sleep superinduced by washing out the stomach. And not only has temporary relief been thus secured but permanent cure has followed the proper and judicious use of this remedy. It removes all small decomposing débris and sources of irritation, and allows nature to reassert itself, and thus relieve the constant fretting and irritability of the stomach. Following this come the proper non-irritating tonics and due regard to systematic, judicious feeding.

DR. McHATTAN thinks that Dr. Gewinner has struck the key-note in nine-tenths of the diseases of infancy and childhood. If he was to use one word as the probable source of a very large per cent. of the diseases of this period of life, that word would be *dieting.* He would take issue with Dr. Gewinner in the use of pilocarpine in uremic troubles; he had been unfortunate in its use in these troubles; he referred to the words of some prominent author who said that the usual results of this drug in this class of diseases was that the patient "revives, brightens up, makes his will and dies"—and such had been his experience with it, though he had used it only in extreme cases.

DR. FERGUSON indorses the views expressed in the paper, and by the gentlemen who had spoken. For twenty-five years he had maintained that Nature has given us an indication as to the proper feeding by the process of dentition, and until the teeth are all through, and the child had been thus prepared for mastication, he had always taken the position that nothing but the most easily digested articles should find their way into the stomachs of these little people.

DR. GEWINNER, in closing the discussion, said that he would mention one point to which he had not given sufficient emphasis in his paper, viz., the proper intervals of feeding, either from the breast or artificial feeding. In proportion to the age of the child should the intervals be regulated, and sufficient time should be allowed for the digestion of one nursing before another is allowed. And *this* in a large measure should be regulated by the condition of the alimentary tract in each individual case at the time the instructions are given.

